# Streamlining CRISPR spacer-based bacterial host predictions to decipher the viral dark matter

**DOI:** 10.1093/nar/gkab133

**Published:** 2021-03-02

**Authors:** Moïra B Dion, Pier-Luc Plante, Edwige Zufferey, Shiraz A Shah, Jacques Corbeil, Sylvain Moineau

**Affiliations:** Département de biochimie, de microbiologie et de bio-informatique, Faculté des sciences et de génie, Université Laval, Québec City, Québec G1V 0A6, Canada; Groupe de recherche en écologie buccale, Faculté de médecine dentaire, Université Laval, Québec City, Québec G1V 0A6, Canada; Centre de recherche en infectiologie de l’Université Laval, Axe maladies infectieuses et immunitaires, Centre de Recherche du CHU de Québec-Université Laval, Québec City, Québec G1V 4G2, Canada; Centre de recherche en données massives, Université Laval, Québec City, Québec G1V 0A6, Canada; Département de médecine moléculaire, Faculté de Médecine, Université Laval, Québec City, Québec G1V 0A6, Canada; Département de biochimie, de microbiologie et de bio-informatique, Faculté des sciences et de génie, Université Laval, Québec City, Québec G1V 0A6, Canada; Groupe de recherche en écologie buccale, Faculté de médecine dentaire, Université Laval, Québec City, Québec G1V 0A6, Canada; COPSAC, Copenhagen Prospective Studies on Asthma in Childhood, Herlev and Gentofte Hospital, University of Copenhagen, Gentofte 2820, Denmark; Centre de recherche en infectiologie de l’Université Laval, Axe maladies infectieuses et immunitaires, Centre de Recherche du CHU de Québec-Université Laval, Québec City, Québec G1V 4G2, Canada; Centre de recherche en données massives, Université Laval, Québec City, Québec G1V 0A6, Canada; Département de médecine moléculaire, Faculté de Médecine, Université Laval, Québec City, Québec G1V 0A6, Canada; Département de biochimie, de microbiologie et de bio-informatique, Faculté des sciences et de génie, Université Laval, Québec City, Québec G1V 0A6, Canada; Groupe de recherche en écologie buccale, Faculté de médecine dentaire, Université Laval, Québec City, Québec G1V 0A6, Canada; Félix d’Hérelle Reference Center for Bacterial Viruses, Université Laval, Québec City, Québec G1V 0A6, Canada

## Abstract

Thousands of new phages have recently been discovered thanks to viral metagenomics. These phages are extremely diverse and their genome sequences often do not resemble any known phages. To appreciate their ecological impact, it is important to determine their bacterial hosts. CRISPR spacers can be used to predict hosts of unknown phages, as spacers represent biological records of past phage–bacteria interactions. However, no guidelines have been established to standardize host prediction based on CRISPR spacers. Additionally, there are no tools that use spacers to perform host predictions on large viral datasets. Here, we developed a set of tools that includes all the necessary steps for predicting the hosts of uncharacterized phages. We created a database of >11 million spacers and a program to execute host predictions on large viral datasets. Our host prediction approach uses biological criteria inspired by how CRISPR–Cas naturally work as adaptive immune systems, which make the results easy to interpret. We evaluated the performance using 9484 phages with known hosts and obtained a recall of 49% and a precision of 69%. We also found that this host prediction method yielded higher performance for phages that infect gut-associated bacteria, suggesting it is well suited for gut-virome characterization.

## INTRODUCTION

Bacteriophages (phages: viruses that infect bacteria) are the most abundant and genetically diverse biological entities on the planet. Their ubiquity is equal to that of their hosts’: Wherever there are bacteria, there are phages that can infect them (1). Phages play major roles in their own ecosystems by contributing to bacterial mortality, remodeling bacteria through horizontal gene transfer and rewiring host metabolism (2). Still, phages remain largely understudied compared to bacteria. For example, the number of phage genomes in public databases is still ∼20-fold lower than that for bacteria despite their extensive abundance and diversity. Phages do not possess a conserved gene such as ribosomal RNA in bacteria, making it impossible to detect both known and novel phages using a universal genetic marker, although some have tried (3). In addition, phages are obligate intracellular parasites, hence they require a bacterial host to be propagated, isolated and characterized. This makes isolating new phages challenging when the host has not yet been identified and even impossible when the host is presently unculturable.

Viral metagenomics offers a way to analyze the total viral genomic content in a given sample, circumventing some of the limitations associated with phage studies since it does not require the culturing or identification of hosts (4). High throughput sequencing techniques have drastically accelerated the discovery rate of new phage sequences, either from non-targeted shotgun metagenomics (total DNA) or viral metagenomics (DNA or RNA from the viral fraction). For instance, the most widespread and abundant phages in the human gut (5) and in the oceans (6) were discovered as a result of studies using metagenomics. However, phages are extremely diverse and virologists are confronted with the viral dark matter obstacle: the majority of reads or contigs in viral metagenomes match no known sequences in reference databases (7). Therefore, most of the newly discovered phage sequences lack characterization in terms of morphology, gene content, phylogeny and host range. In the most recent and largest survey of ocean viromes using metagenomics, 338 398 (90%) viral populations could not be taxonomically assigned to a known family (8). Consequently, any additional information on this considerable viral dark matter is necessary to better characterize phage communities and how they interact with their hosts in various ecosystems.

For any novel phage, a fundamental feature is the identification of its bacterial host. There are several bioinformatics approaches that predict the host of a phage [reviewed in (9)], of which overall, the best predictions have been made using sequence homology-based approaches. These approaches involve exact nucleotide matches between the phage and a prophage that is integrated into the genome of its bacterial host, and nucleotide–nucleotide homology of the phage and its host genes. CRISPR (Clustered Regularly Interspaced Short Palindromic Repeats) spacers can also be used for homology-based predictions. Together with *cas* genes, CRISPR loci form CRISPR–Cas systems, an adaptive defense mechanism against invading nucleic acids, such as phage genomes (10–12) and other mobile genetic elements (13). CRISPR–Cas systems are found in ∼40% of all bacterial genomes sequenced to date (10). Bacteria possessing an active CRISPR–Cas system can become resistant to specific phages by incorporating short DNA fragments, called spacers, into their CRISPR locus (11,14). These spacers originate from the invading phage genome and allow the bacterial cell to efficiently recognize and block a subsequent infection from a phage carrying a spacer-matching sequence. Spacer acquisition mostly occurs at the 5′ end of the CRISPR locus, while the 3′ end carries ancestral spacers (15). CRISPR spacers are thus very useful for host predictions since they essentially contain molecular records of past phage infections within the bacterial chromosome and therefore clearly link phages with their hosts. The structure of CRISPR loci also generally represents a chronological history of past interactions between bacteria and phages.

CRISPR spacer-based host predictions however exhibit low sensitivity (∼12.5% for exact matches) and poor accuracy (15%) when there are no appropriate cut-offs (9). The fact that not all bacteria carry a CRISPR–Cas system most likely contributes to the low fraction of predicted phages of this spacer approach. In addition, the precision might be lowered by pervasive horizontal gene transfer of CRISPR–Cas systems (16), which would not always reflect a genuine phage-host interaction. Spacers also undergo a certain level of turnover, leading to spacer loss over time, particularly in the middle of the array. However, this phenomenon has been modeled only a few times and studied very little *in vitro* (17,18). Several programs have been developed to identify CRISPR loci within bacterial genomes (19–22), with CRISPRDetect (23) and CRISPRCasFinder (24) being the most recent. These two programs can predict the 5′–3′ orientation of the locus, which is essential for understanding the chronology of phage infections. Because host predictions using CRISPR spacers are homology-based, they primarily depend on the size of the database against which the new phage sequences are compared. As such, it is very likely that CRISPR spacer host predictions have low sensitivity due to undersampling of the total diversity of available spacers.

The aim of this work was to increase both the recall (percentage of phages that yielded a prediction) and the precision (percentage of accurate predictions) of the CRISPR spacer host prediction approach, to set guidelines to obtain accurate predictions and to explore and analyze its performance for different hosts. We took advantage of the recent progress in CRISPR identification programs to survey CRISPR loci in all bacterial genomes in the NCBI database and increased the number of spacers available for homology searches. Over 11 million spacers were recovered from 367 446 bacterial genomes and they were organized into a spacer database available at http://crispr.genome.ulaval.ca, allowing users to search and download the database in a user-friendly and customizable fashion. Next, we evaluated the recall and the precision of the spacer database by performing a homology search on reference phages with a known host as a benchmark for our studies. The recall and the precision were measured for different alignment metrics, such as the number of mismatches and the *E*-value. Finally, we set up a decision tree of logical rules inferred from phage biology to improve the precision of the predictions. For predictions with at most two mismatches between a spacer and a matching viral sequence, we obtained predictions for 49% of phages and a precision of 69% at the genus level.

## MATERIALS AND METHODS

### Spacers in bacterial genomes

All bacterial genomes in the NCBI database (*n* = 580 383) were downloaded on 23 March 2020 in FASTA format using the file transfer protocol. The file path for each genome was obtained from the assembly_summary.txt file (ftp://ftp.ncbi.nlm.nih.gov/genomes/genbank/bacteria) under the ‘ftp_path’ column. We then executed the command-line version of CRISPRDetect v2.2 to identify CRISPR loci in these bacterial genomes using an -array_quality_score_cutoff of 3 as recommended for FASTA files. The GFF file generated by CRISPRDetect was parsed to extract spacers (a row corresponds to a spacer when the feature type in the third column is ‘binding_site’) and the following metadata for each spacer: accession number of the bacterium (first column), start and end positions on the genome (fourth and fifth columns), length (sixth column), orientation (seventh column), locus number and position inside the locus (ninth column after ‘ID = ’) and sequence (ninth column after ‘Note = ’).

### SQL spacer database, web platform and command-line tool

An Sqlite database was constructed using Python v3.8.5 and the Sqlite3 v2.6.0 module. For each genome, the species, genus, family, order and suborder were retrieved using the Python ete3 v3.1.1 module (25) which interacts with the NCBI taxonomy tree. Due to recent updates in the bacterial taxonomy database that have yet to be reflected in GenBank, some entries were manually searched online in the NCBI taxonomy database and incorporated into the spacer database. The spacers for each bacterial genome were retrieved from the GFF files that were produced by CRISPRDetect and inserted in the Sqlite database. The complete code that was required to reproduce these steps is available with the phage host prediction tool on GitHub (http://github.com/edzuf/CrisprOpenDB). A web application was created to permit database exploration and to allow experimentation with the phage host prediction tool. The application is a Plotly Dash application implemented with Python. This web application hosts a complete version of the Sqlite database that can be downloaded as CSV or FASTA files. A command-line host prediction tool was also implemented using Python. The main purposes of this tool were to allow users to run predictions for large numbers of phage genomes and to offer a more customized host prediction process. The tool supports multi-FASTA files that contain all phage genomes for which a host prediction must be computed. The alignment step with blastn already offers the option to set the desired number of threads. Computation times were therefore already optimized for this step. However, information retrieval from the spacer database and host prediction for each genome required parallel programming to increase speed, so we used the Python ‘multiprocessing’ package. Several tests were carried out to assess the speed of the command-line tool. Six different-sized datasets (10, 50, 100, 500, 1000 and 5000 genomes) were created. For each size, 10 subsets of phages were randomly generated. For each subset, the host prediction command-line tool was executed, and the computation time was measured. The complete code and instructions for installing the command line tool are available on GitHub (http://github.com/edzuf/CrisprOpenDB). A version of the Sqlite database must be downloaded in order to run the predictions locally.

### Benchmark phage dataset

All phage genomes (*n* = 12 737) used in this study were retrieved from the NCBI Virus database (26) on 23 April 2020, using the following filters: Virus = ‘Bacteriophage, all taxids’ and Nucleotide completeness = ‘complete’. This list included several duplicates, mostly due to phage genomes being available in both RefSeq and GenBank databases. These duplicates were relatively easy to identify as they had the same GenBank Title. One occurrence of each of these duplicate genomes was kept. Phage genomes that were deposited after experimental evolution experiments were also excluded, as they could lead to overrepresentation of some sequences. These were recognized by the presence of the words ‘mutant’, ‘clone’, ‘isolate’ or ‘evolved’ in the GenBank Title. In addition, phages with either a non-bacterial or unknown hosts were removed. This is caused by inconsistencies in the nomenclature of phage genomes, especially those identified through metagenomics. This resulted in a total of 9484 phage genomes (see Supplementary dataset 1 for the complete list). The hosts of these phages were inferred from the ‘Host’ column, or when empty, from the GenBank Title (e.g. *Escherichia* is the host of Escherichia phage Lambda).

### Optimization of pipeline for host prediction

To predict the hosts of these phages using a CRISPR-based method, we searched for homology between the spacers and the phage genomes. A multi-FASTA file was created with all the spacers in the spacer database that ranged from 23 to 48 nucleotides (*n* = 11 674 395). Blastn v2.2.26 (27) was then used to perform nucleotide-nucleotide pairwise alignments with the -task ‘blastn’. Hits were saved in a tabular format and exported in a Python 3 Jupyter Notebook for further analyses. For each hit between a spacer and a phage, a predicted host was assigned based on the bacterial origin of the spacer. We evaluated the recall and the precision of different *E*-values, and numbers of mismatches to determine the cut-off that recovered the highest number of host predictions while still preserving the best accuracy in terms of predicted hosts. The *E*-value was automatically computed by the alignment program. The true number of mismatches was calculated as: (spacer length – alignment length + number of mismatches computed by blastn). All hits with gap openings were discarded because the presence of gaps makes it unworkable to calculate the true number of mismatches. The precision of the prediction was measured at different bacterial taxonomic levels by computing the rank of the last common ancestor (LCA) between the biologically confirmed and the predicted hosts. The lineages for both real and predicted hosts were obtained with ete3 by first converting the host bacterial names into taxids with the NCBITaxa.get_name_translator() function, then by translating the taxids into a hierarchically sorted list of parent taxids with the NCBITaxa.get_lineage() function. The two lineages were compared to identify the LCA and its rank was obtained with the NCBITaxa.get_rank(). Depending on the cut-off, numerous spacers that originated from different bacterial genera matched the same phage. Two different approaches were applied sequentially to determine which predicted host was the most likely to be the real host. In the first step, the bacterial genus with spacers that targeted the highest number of different regions in the phage genome was considered the predicted host. This was deduced from the start and end positions on the phage genome of each alignment with a spacer. In the second step, for each genus that was predicted to be the host of the same phage, we calculated the relative position for all the spacers that matched the phage genome within the CRISPR locus. We then considered the genus with the spacer that was closest to the 5′ end of its locus to be the predicted host as the most recently acquired spacers are usually at the 5′-end of a CRISPR array. The relative position is a transformation of the absolute position within the locus of the spacer. This ensures that all spacers have a numerical position within the same range (0 to 1), despite various lengths of CRISPR loci. If the prediction still yielded multiple hosts (with equal highest number of targets on the phage genome and closest spacers to the 5′ end), the predicted host was their LCA.

### Statistics

A null model was generated to evaluate whether the cut-offs and filters that we proposed yielded better predictions than selecting a random host from the alignment hits. For each phage, a random hit (spacer) was selected from the blastn results and the corresponding bacterium was assigned as the randomly predicted host. For predictions obtained with the first filter, random hits corresponded to any hits from the raw blastn results, irrespective of the number of mismatches. For predictions obtained with the second filter, random hits consisted in selecting a random host from all hits that passed the first filter. For the third fiter, the same logic was applied, except that we selected a random host from all hits that passed the first and second filters. The random prediction was considered successful when the randomly predicted host was identical to the real host at the genus level. The success rate was measured as the percentage of phages for which the host was successfully predicted at random. This measurement was performed for 1000 simulations.

### Proof of concept on a published gut virome

We tested our host prediction pipeline on a recently published dataset of human gut viromes (28). After a first round of decontamination, the authors obtained 57 721 contigs. We used VIBRANT v1.2.1 (29) to further curate the sequences of these same contigs and confirm they were of viral origin. Contigs that were confirmed to be viruses by VIBRANT were then used to explore the breadth of bacterial hosts that were predicted for this dataset. A specific phage contig (metaspades_NG-13376_921T3_lib202033_5478_NODE_37_length_22057_cov_6.42255) was examined for the presence of an integrase or a recombinase, indicating it may be temperate. We searched for ‘recombinase’ and ‘integrase’ in the phage contig annotation performed by VIBRANT. Additionally, hmmsearch v3.3 (30) was executed to search for a large serine recombinase in the phage contig predicted proteins, using the hmm profile of the protein family resolvase (PF00239).

### Comparison with an existing host prediction method

We compared the recall and the precision of our approach with WIsH v1.0 (31), a program based on homogeneous Markov models to predict potential bacterial hosts. The authors used 3780 bacterial genomes and 1420 phages to evaluate the accuracy of their predictions. All 1420 phages were part of our benchmark dataset, except for NC_003525 (host is unknown) and NC_020836 (no longer exists). To evaluate performance, we measured the recall and the precision for the 1418 phages using our host prediction approach and compared our results with those obtained in the previous study using WIsH.

## RESULTS

### The spacer database represents an unprecedented catalogue of spacer diversity

**Figure 1.**
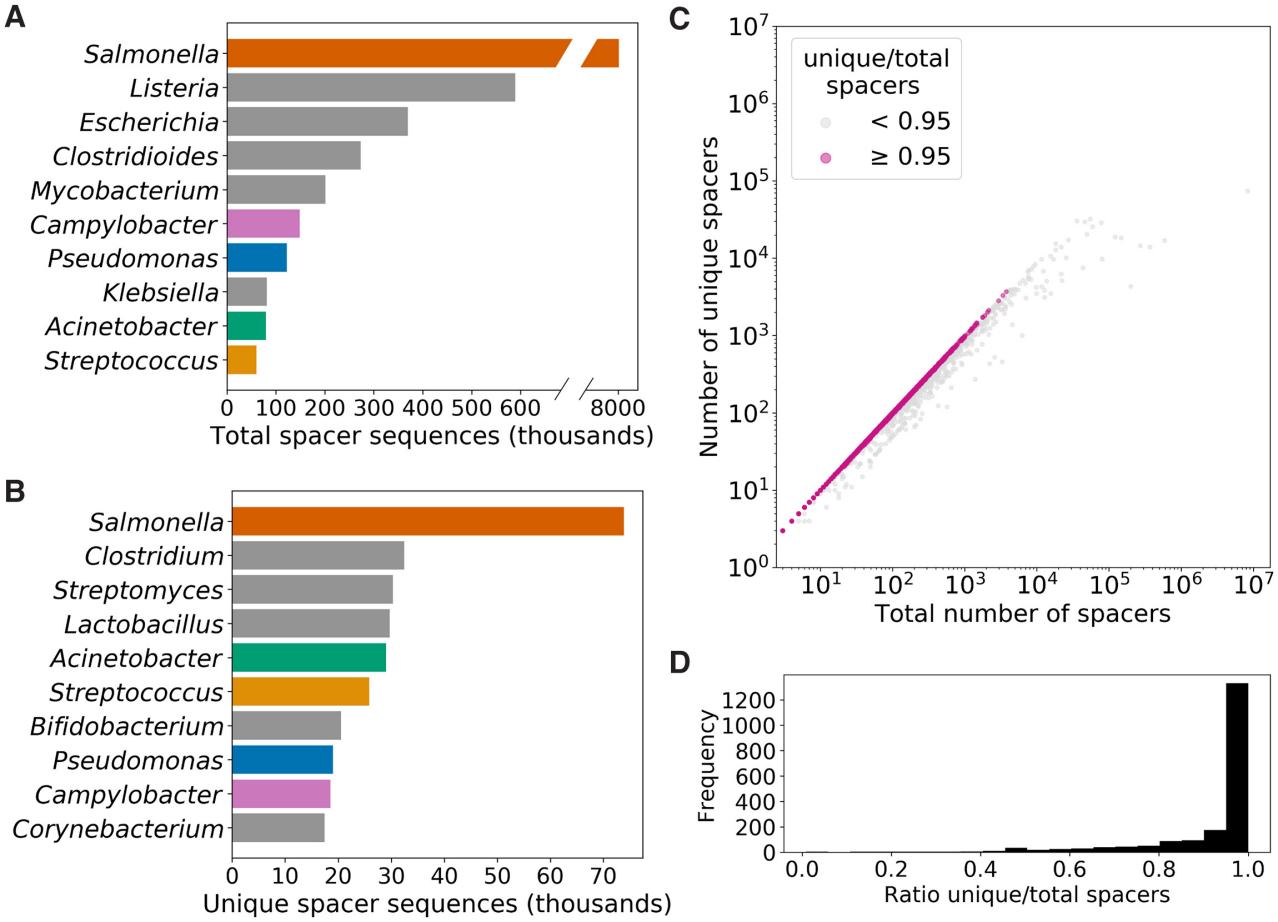
Overview of the spacer database diversity. (**A**) Total number of spacers and (**B**) number of unique sequences from the top 10 bacterial genera in the spacer database. Coloured bars represent genera that appear in the top 10 for both total and unique sequences. (**C**) Total number of spacers and number of unique spacers for each bacterial genus in the spacer database (each marked by coloured dots). Grey and pink dots represent ratios of unique versus total spacers that are smaller or greater than 0.95, respectively. (**D**) Distribution of the ratio of unique vs total spacers.

We used CRISPRDetect to identify CRISPR loci in all bacterial genomes in the NCBI database. Out of 580 383 genomes available at the time of the analysis, we identified at least one CRISPR locus in 367 446 of them (63.3%). This represents a total of 11 767 782 spacers, ranging from 1 to 419 nucleotides. Since spacers ranging from 28 to 43 nucleotides represent 99.2% of all sequences, spacers outside this range likely represent errors introduced by CRISPRDetect, either from erroneously splitting the repeat extremities from the complete repeat sequence (leading to a short spacer), or from not distinguishing repeats from spacers (resulting in a long spacer). These were removed from further analyses but are still available in the online database. The spacers belonged to 1978 bacterial genera and the most widely represented hosts were strongly influenced by the number of genomes available in the NCBI database. Accordingly, spacers belonging to *Salmonella* were by far the most frequently identified (*n* = 8 325 687), followed by *Listeria* (*n* = 588 364) and *Escherichia* (*n* = 368 069) (Figure 1A). There is a total of 1 313 992 unique spacer sequences, indicating a high level of sequence redundancy among the 11 million spacers. The genus *Salmonella* also had the highest number of unique spacers (*n* = 73 797), whereas *Escherichia* and *Listeria* were not even in the top ten (11th and 15th positions, respectively) (Figure 1B). Similarly, *Clostridium* was the 11th most represented genus in the total number of spacers but had the second highest number of unique spacers (*n* = 32 375). However, for the ratio of unique/total spacers (Figure 1C), the genus *Salmonella* had the smallest ratio (0.009). The case of *Salmonella* is a reminder that using the complete GenBank database adds considerable redundancy in our spacer database for heavily sequenced organisms with less diverse CRISPR arrays. In contrast, most of the genera (67%) examined had a ratio that was ≥0.95 (Figure 1D), meaning that almost all of their spacers have unique sequences.

### A website and command-line tool for exploration and high-throughput predictions

**Figure 2.**
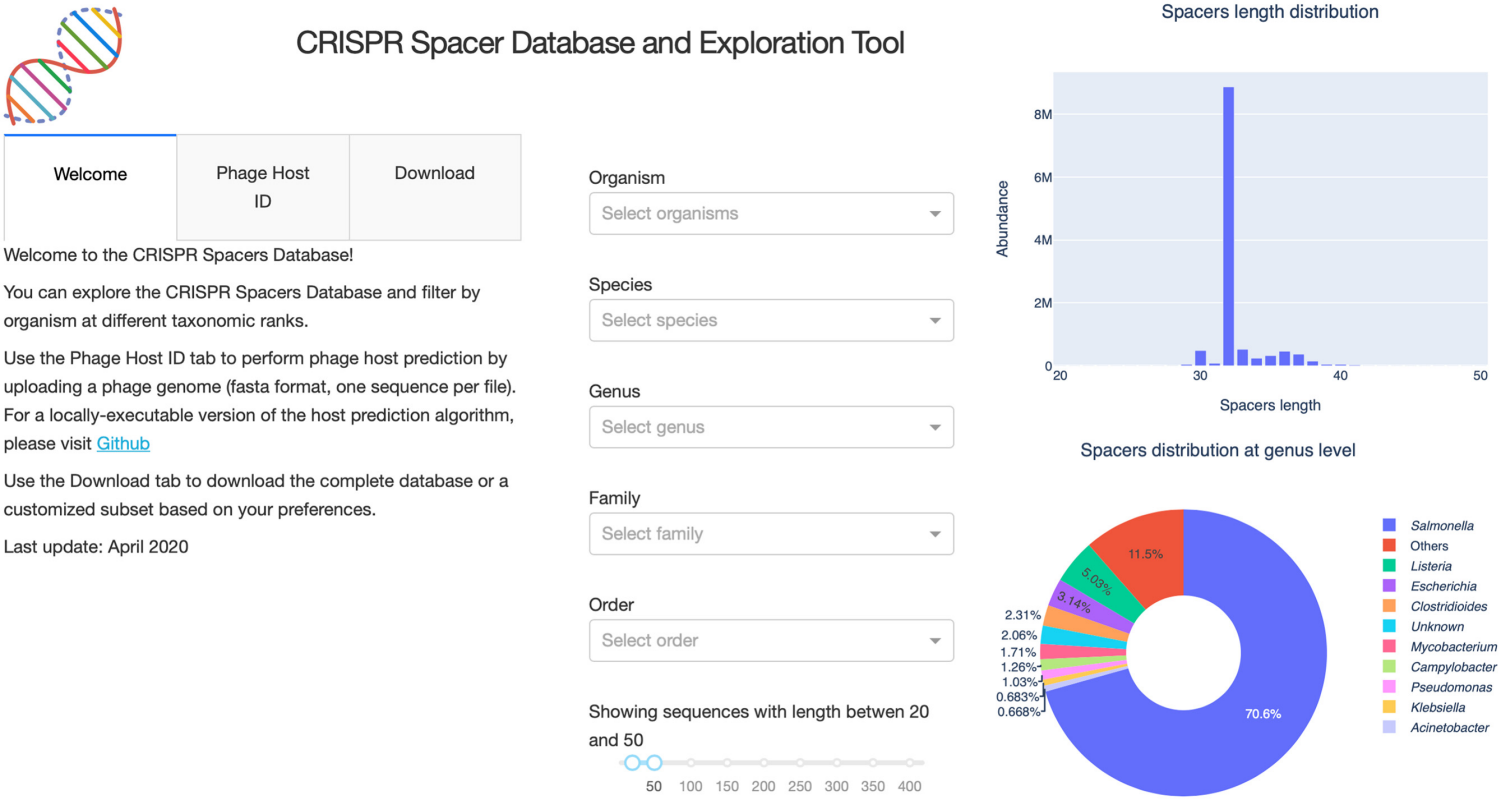
Overview of the spacer database website (http://crispr.genome.ulaval.ca). The homepage features dropdown menus that filter spacers according to the organism at different taxonomic levels. It also presents summary figures that represent the distribution of spacer sizes and number of spacers per organism.

**Figure 3.**
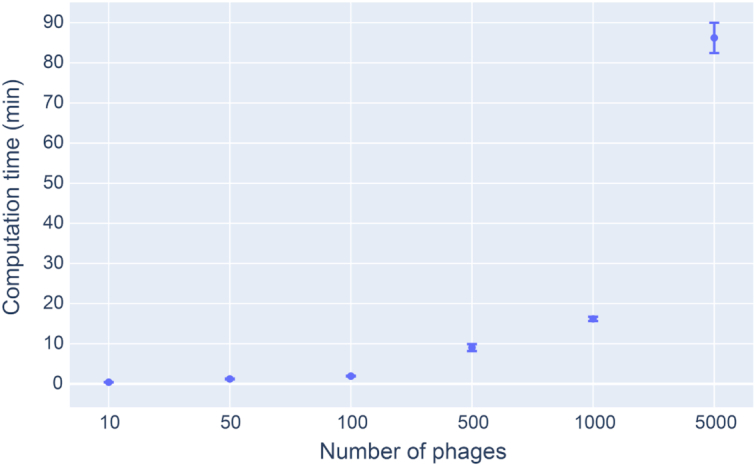
Average host prediction computation time for datasets comprising 10–5000 phage genomes. Phage genomes were randomly selected from the complete dataset of 9484 phages.

A web platform (Figure 2) was developed with the objective of sharing the spacer database with the scientific community. To navigate across the different bacterial genomes and spacers, searchable dropdown menus allow the user to filter by species, genus, family and order. The spacers can be displayed in a table that is linked to the NCBI Organism database. The phage host identification tool used in conjunction with the spacer database can be tested online. Single genome FASTA files can be uploaded to the application and a host prediction will be made. The server only permits the use of default parameters in order to deliver the fastest and best results. Custom parameters can be used by installing the phage host identification command-line tool from GitHub (https://github.com/edzuf/CrisprOpenDB). The command-line tool is a Python package that can be used for batch processing. It is not limited in the number of genomes or by the genome length that can be processed. From start to finish, the program took on average 86 min to perform host predictions on a dataset of 5000 phages, running on 32 CPUs (Figure 3). A full host-prediction report can be produced and explains each step of the analysis while providing the list of spacers on which a prediction is based. For a deeper understanding of a specific prediction, a table containing both alignment results and spacer information extracted from the database can be generated and saved as a CSV file. The list of all customizable parameters implemented is shown in Supplementary Table S2.

### Systematic filters increase phage host prediction performance

**Figure 4.**
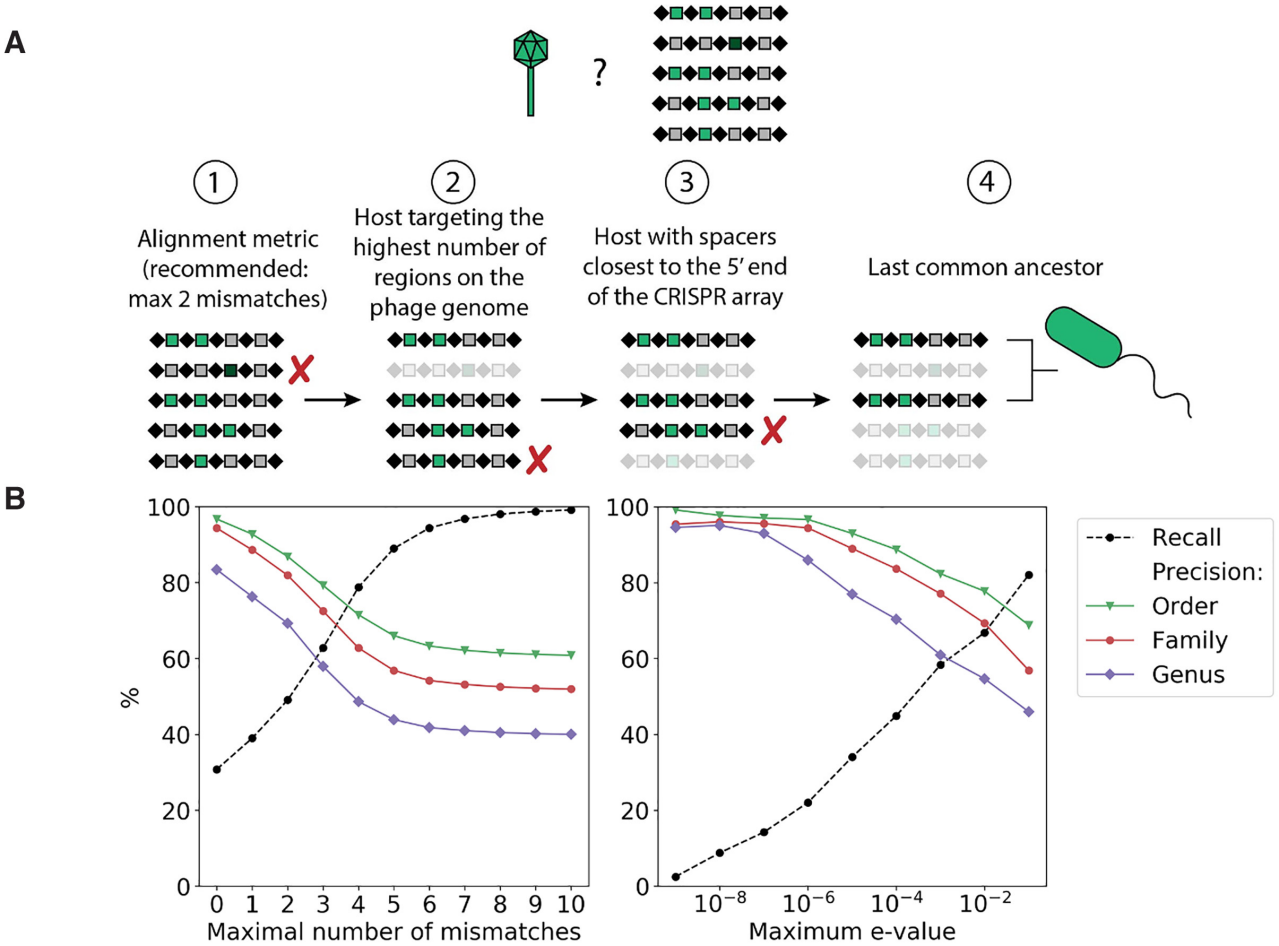
A systematic approach for predicting the bacterial host of unknown phages. (**A**) Four criteria were sequentially applied to discard false positives from the spacer-phage alignment results. These criteria were inspired from the way the CRISPR–Cas systems work as an adaptive immune system and are biologically relevant. (**B**) Recall (dashed line) and precision at three taxonomic levels (solid line; red represents genus, green represents family, blue represents order) of the host predictions when the number of mismatches (left) or the *E*-value (right) was used as the first filter.

We used a set of criteria and filters to select the most probable bacterial host for unknown phage sequences (summarized in Figure 4A). These filters were essential considering the high level of redundancy and bias in the number of spacers found in bacterial genera for which numerous genomes have been sequenced. Without proper filters, selecting a random host from the alignment result would be greatly influenced by these biases. The filtering conditions have biological meanings and are data driven, which helps to make our prediction algorithm easier to interpret. The performance of the implemented filters was tested using 9484 phage genomes with known hosts from the NCBI Virus database. After searching for sequence homology between the phage genome and the spacer database using blastn, four filters were applied sequentially. During the first step, we evaluated the performance of two different alignment metrics to use as filters to discard inaccurate alignment hits. Specifically, we examined the number of mismatches (0–10) and the *E*-value (10^–9^–10^–1^). The number of nucleotide mismatches reflects the number of mutations between a spacer in the CRISPR locus and a protospacer in the phage genome and hints at the elapsed evolutionary time since a phage and its host last interacted. For the number of mismatches specifically, hits with the least number of mismatches within the cut-off limit are kept. Following this first step, if the remaining spacers belonged to the same bacterial genus, that genus was assigned as the predicted host of the phage. Otherwise, up to three additional filters were applied. Similarly, if only one bacterial genus was found for every additional filter, it was assigned as the host of the phage and the prediction algorithm ended. In the second step, only bacterial genera that targeted, through their spacers, the highest number of different regions on the phage genome passed through the filter. The rationale behind this step is that hosts with spacers targeting multiple regions of the phage genome are more likely reflecting genuine phage–host interactions, compared to hosts targeting single regions, perhaps caused by random hits. During the third step, only bacterial genera whose spacers were closest to the 5′ end (leader end) in their corresponding CRISPR array were retained. Similar to the first filter, this third filter provides a measure of time since the last phage–host encounter, because new spacers are incorporated at the 5′ end of the array. Thus, spacers closer to the 5′ end are more recently acquired compared to those at the 3′ end. For the fourth and final step, if all previous filters and conditions failed to identify a single host genus, the last common ancestor of the remaining bacterial genera was considered to be the phage host.

The recall and the precision at three taxonomic bacterial ranks (genus, family and order) for different numbers of mismatches and *E*-values are shown in Figure 4B. Both the number of mismatches and the *E*-value yielded similar results: strict cut-offs (low numbers of mismatches or low *E*-values) provided more accurate predictions but were less sensitive, whereas more relaxed cut-offs increased the recall and reduced the precision. The overall highest precision reached 95% at the genus level and was obtained with an *E*-value of 10^–9^. This was however accompanied by a very low recall of only 2.5%. A zero-mismatch cut-off reached a precision of 84% at the genus level and a recall of 31%. In general, for a given recall, using the number of mismatches resulted in a higher precision by at least 5% than when the *E*-value was used. Although the *E*-value yielded good performance, we did not retain it for two reasons. First, *E*-values depend on the length of the query (phage genome) and the size of the database. Both can vary substantially, as phage genomes have a wide range of sizes, and because the database size will inevitably increase when updated over time. Second, *E*-values are harder to interpret biologically and setting a threshold for the *E*-value would be somewhat arbitrary. In the context of CRISPR–Cas systems, the number of mismatches is a biologically meaningful alignment metric since Cas interference complexes vastly rely on spacers to be identical to the invading nucleic acids in order to efficiently recognize and cleave it. To maximize both the recall and the precision, we thus recommend a maximum tolerance of two mismatches, which yielded a precision of 69% and a recall of 49%. By tolerating two mismatches, we allow for sequencing errors or mutations without compromising the precision too greatly. The optimal order of the filters was also determined after testing all possible combinations and selecting the order which conferred the best compromise in recall and precision (Supplementary Table S1). The number of mismatches necessarily needs to be the first filter for the two other filters to perform accurately, otherwise they may be applied on hits with many mismatches.

### Relevance of each filter in obtaining high-quality predictions

**Figure 5.**
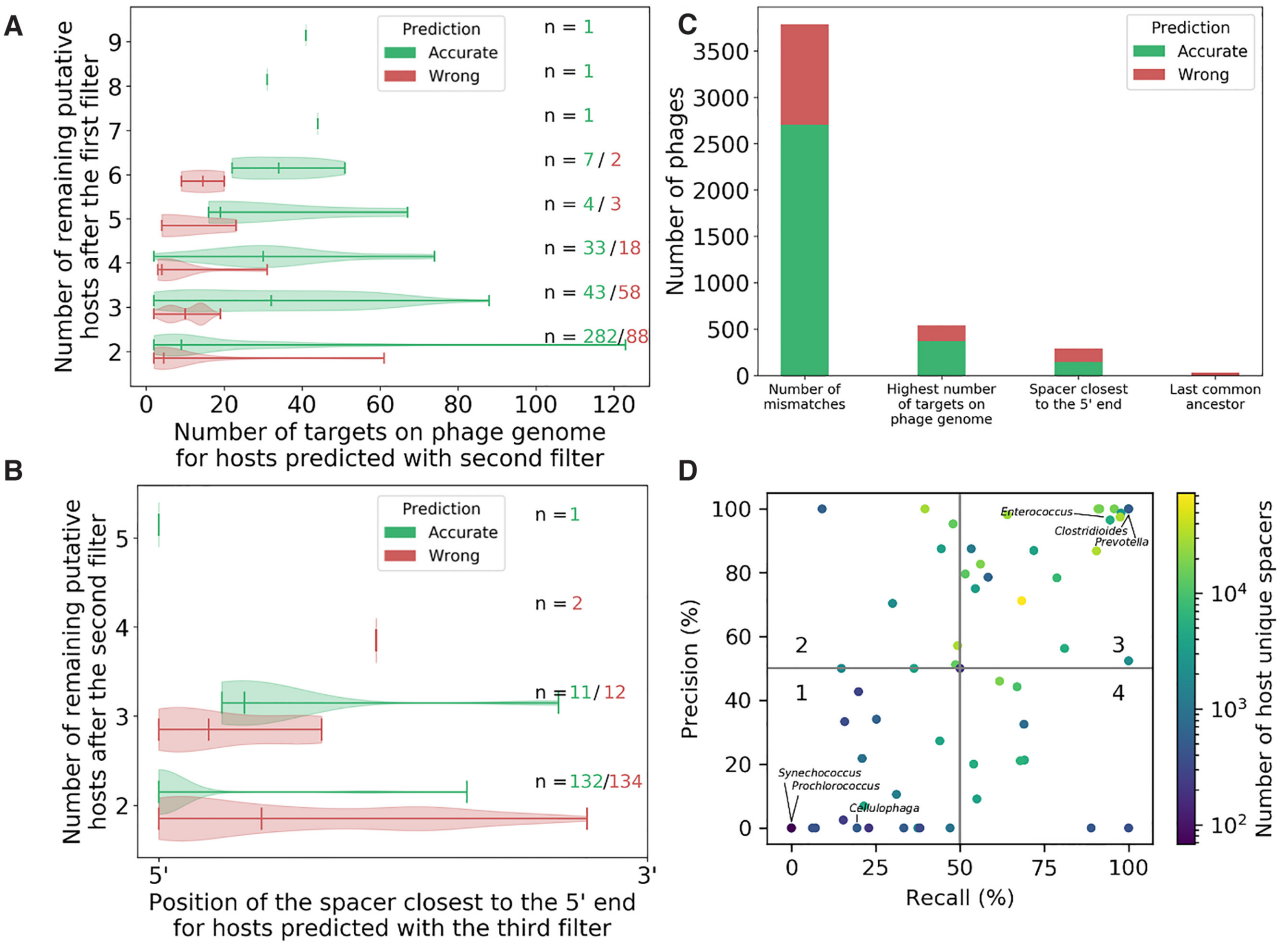
Contribution of each filter and effect of the host on prediction performance. (**A**) Violin plot of the number of targets on the phage genome for hosts predicted with the second filter and number of hosts that passed through the first filter. Accurate predictions are shown in green and inaccurate predictions are shown in red. (**B**) Violin plot of the position of the spacer closest to the 5′ end of the CRISPR array for hosts predicted with the third filter. Accurate predictions are shown in green and inaccurate predictions are shown in red. (**C**) Number of accurate (green) and wrong (red) predictions when either one of the four filters yielded a prediction. (**D**) Recall and precision for groups of phages that infect the same bacterial host (each represented by a dot). The graph is separated into four categories based on the recall and the precision values (higher or lower than 50%).

To confirm that our selected criteria translate into accurate predictions, we compared the distribution of accurate and wrong predictions according to the host's number of targets on the phage genome (for hosts predicted following the second filter) or the host's spacer position in the array (for hosts predicted following the third filter) (Figure 5A and B). A total of 541 phage host predictions were obtained by applying the second filter. Most of these phages (*n* = 370, 68%) had two putative hosts following the first filter, but we found three phages that had more than six predicted hosts remaining after the first filter. These three phages all infect *Klebsiella* and their host was accurately predicted (the other putative hosts were all Enterobacterales). We were also surprised to find five phage genomes targeted at >100 regions by their accurately predicted *Moraxella* host spacers. When comparing accurate and wrong host predictions obtained with the second filter, a clear distinction could be observed in the number of spacer targets on phage genomes. Accurate hosts target a higher number of viral regions, irrespective of the number of putative hosts remaining after the first filter, which confirms the relevance of this criterion (Kolmogorov–Smirnov statistic = 0.38, *P*-value = 2.00 × 10^–15^). Next, the third filter was responsible for a total of 292 phage host predictions. At this point, phages are left with less putative hosts, with the majority having two predicted hosts remaining after the second filter (*n* = 266, 88%). There were three phages with more than three hosts (phages that infect *Bordetella*, *Actinomyces* and *Burkholderia*). For phages with two remaining putative hosts after the second filter, there was a difference in the distribution of spacer position for accurate and wrong predictions. Hosts with phage-matching spacers closest to the 5′ end led to more accurate predictions, consistent with our biological explanation (Kolmogorov–Smirnov statistic = 0.59, *P*-value = 5.55 × 10^–16^). We however found a reverse trend for phages with three remaining putative hosts after the second filter, but these represent only 8% of the predictions obtained from the third filter. This is a good reminder that the spacer acquisition rate between organisms is different and might be the reason why this third filter is erroneous in some contexts.

We next examined how the accuracy varied across filters (Figure 5C). The first and second filters contributed the most to singling out a host, as together they represented 93% of all predictions made. The fourth filter (last common ancestor), intended as a safety net in case previous filters failed, was rarely employed (<1%) for making a prediction. The first and second filters also showed the highest accuracy, as their predictions had 71 and 69% precision, respectively. The third filter (spacers closest to the 5′ end of the CRISPR array) yielded less precise predictions (49% precision) and represented 6% of all predictions. Despite various levels of precision, the three filters systematically performed better than selecting a random host (null model, *P*-value < 0.001). This was not surprising for the first filter (number of mismatches) because there are several spurious hits from a blastn alignment. However, this confirms the importance of the two additional filters as they improved the quality of the predictions, when compared to selecting a random host from the hits that passed the first filter.

### Host effect on prediction accuracy

Given the variability in the content of various CRISPR arrays, which is linked to the adaptive activity of CRISPR–Cas systems, we suspected that our approach may show different performances depending on the host of the phage. There were 81 cases where the real host was in a tie with other putative hosts after the first filter and led to an inaccurate prediction after the second filter. Of these, 61 were phages infecting *Escherichia*, but were systematically predicted to infect *Salmonella*. This may be caused by broad host range phages classified as Escherichia phages but capable to infect other *Enterobacteriaceae*. Similarly, there were 52 cases where the real host was in a tie with other putative hosts after the second filter and was not picked as the predicted host by the third filter. Here, 19 of these were phages infecting *Mycolicibacterium* and were incorrectly assigned to *Mycobacterium*.

Overall, the performance of the prediction algorithm was found to be host dependent. This is most likely caused by differences in the diversity of spacers for each host, as we found a correlation between the precision and the number of host unique spacers when phages were grouped by their hosts (Figure 5D, Spearman correlation coefficient = 0.69). Prediction performances (based on bacterial host) were separated into four categories, depending on their recall and precision (higher or lower than 50%). We examined the results for categories 1 (low recall/low precision) and 3 (high recall/high precision), as they represented the two largest categories. Interestingly, we found several marine bacteria in category 1 and many gut-associated bacteria in category 3. No host predictions were made for phages that infect *Prochlorococcus* and *Synechococcus* (category 1). Less than 20% of *Cellulophaga* (category 1) phages were predicted but these predictions were systematically inaccurate. These inaccuracies however did not apply to phages that infect *Vibrio*, another marine bacterium, as the recall and precision for this group of phages reached 52 and 70%, respectively. It was previously reported that neither *Synechococcus* nor *Prochlorococcus* harbors a CRISPR–Cas system (32). Consistent with those findings, our spacer database does not contain any spacers that belong to *Prochlorococcus*. There are, however, 2125 spacers that originate from *Synechococcus*. On the other hand, phages that infect *Clostridioides*, *Enterococcus* and *Prevotella* (category 3) could be perfectly (100% recall and precision) or near perfectly associated to their hosts. Other gut bacteria found in category 3 included *Salmonella*, *Proteus*, *Clostridium* and *Bifidobacterium* (Supplementary dataset 2). The incidence of CRISPR–Cas systems was previously reported to be associated with particular ecological traits, such as specific ranges of oxygen levels and temperatures, which might explain why host predictions based on CRISPR spacers are better suited for gut phages than for aquatic phages (33,34).

### A CRISPR spacer-based host prediction method to uncover phages that infect the most dominant bacterial members of the gut

**Figure 6.**
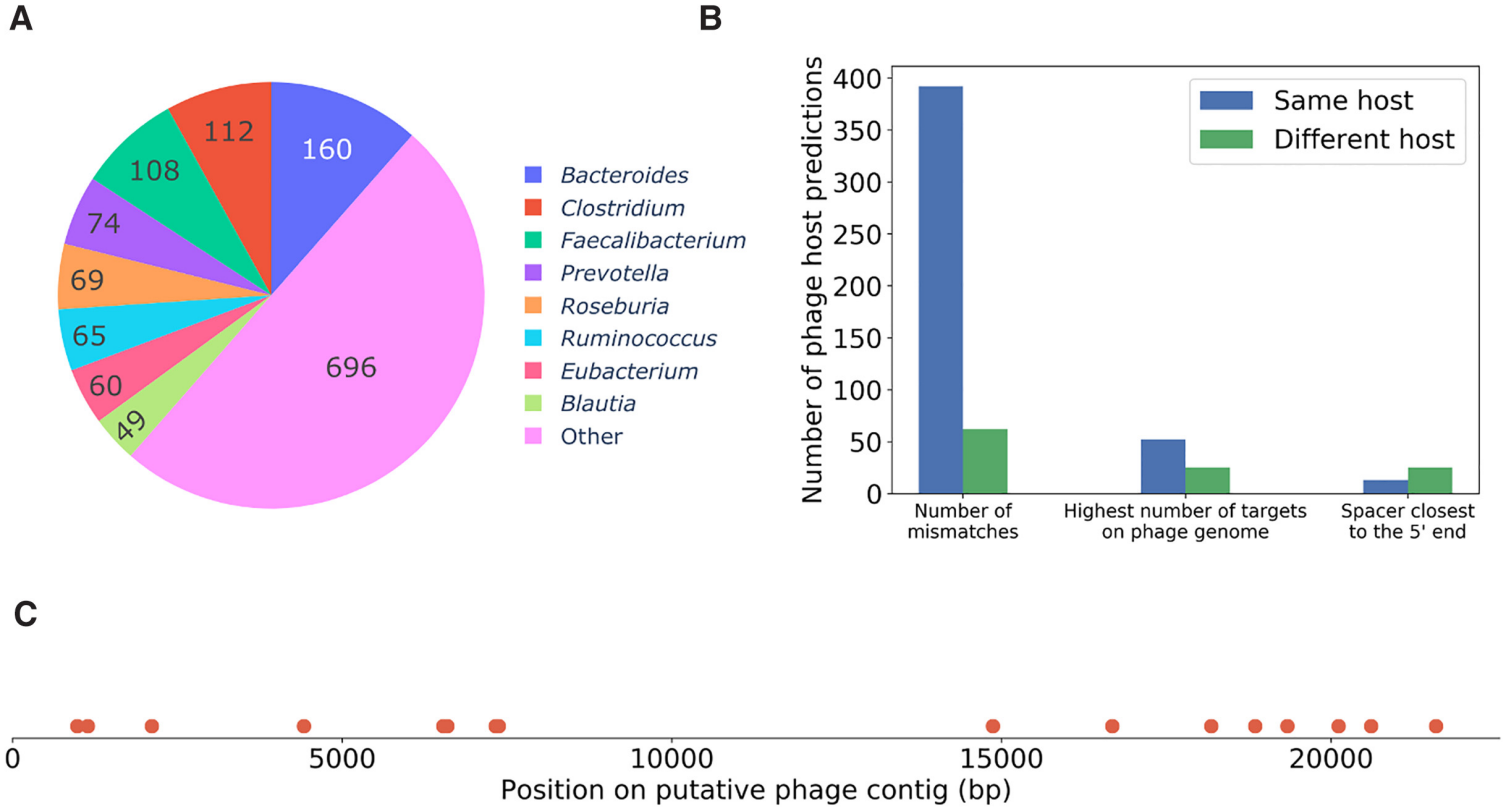
Bacterial host predictions for unknown phages from the gut virome. Eight dominant members of the gut microbiota represent half of the predictions. (**A**) Number of phages predicted to infect each bacterial genus. (**B**) Number of predictions obtained from each filter for gut virome phages when a host was also predicted in the previous Shkoporov study. Predictions that are identical or different to the Shkoporov study are in blue and green, respectively. (**C**) Regions (red dots) on a putative *Clostridium-*infecting phage contig targeted by *Clostridium* spacers.

To illustrate the types of results that can be obtained, we used the recent gut virome study by Shkoporov and colleagues (28) and predicted the bacterial hosts of their discovered phages. Of the initial catalogue of 57 721 contigs, we used VIBRANT to further identify contigs with a viral origin. VIBRANT predicted only 5496 contigs (9.5%) as viral. We predicted the hosts for 1393 phage contigs (25%), with eight bacterial hosts representing half of the predictions (Figure 6A). These top hosts are all dominant members of the human gut microbiota (35), which supports the validity of the predictions. The authors of the initial study had also performed CRISPR spacer-based host predictions, using different genomic sources and software. Of the 5496 viral contigs, they obtained a host prediction for 834 of them. Our approach yielded 67% more predictions. There were 569 viral contigs for which a host was predicted at the genus level from both the initial study (hereby called Shkoporov host predictions) and our present work and after comparing predictions for the same viral contig, we found that 457 (80%) were identical (Figure 6B). The filter used in our prediction approach strongly associated with the two methods giving identical or different predictions. Indeed, identical predictions were made in the vast majority (392/457, 86%) with the first filter (number of mismatches), whereas cases with different predictions were obtained from the first filter only 55% of the time. Thus, cases of conflicting predictions between the Shkoporov host prediction and our method represent more complex decisions, requiring the second and third filters.

We then focused our attention on a phage contig predicted to infect *Clostridium*. This 22-kb contig was targeted by 16 different *Clostridium* spacers (with 0 mismatch) all across the contig (Figure 6C). Shkoporov and colleagues identified this phage as a *Siphoviridae* but were unable to predict its host. Although this phage contig seemed incomplete, no integrase or recombinase was identified, suggesting it may be virulent, whereas most *Clostridium/Clostridioides* phages in the literature are currently temperate (36). Using a combination of viral metagenomics and our host prediction method may provide a means to identify new phages and help isolate virulent phages for various applications. This combination of techniques was proven successful for the isolation of crAssphage, which was initially identified through metagenomics (5), predicted to infect *Bacteroides* and then, isolated four years later with *Bacteroides intestinalis* as its host (37).

### Comparing the CRISPR-based approach with existing methods

We also compared the performance and ease of use of our approach with that of WIsH (31), a host prediction method based on homogeneous Markov models. To compare the recall and the precision, we used the same dataset of 1420 phage genomes from Galiez *et al.* (31). Using our method, we obtained a recall of 17% and a precision of 71%. The precision associated with WIsH is inversely proportional to the number of bacterial genera used to train homogeneous Markov models. Galiez *et al.* (31) used 965 bacterial genera to test the accuracy, whereas our spacer database contained spacers belonging to 1978 bacterial genera. For an equivalent recall of 17%, WIsH precision with 965 bacterial genera reached 80%. There was however insufficient data to precisely evaluate what the recall and precision of WIsH would be with 1978 bacterial genera. We estimated that the relationship between the number of bacterial genera and the precision was best described by a logarithmic function, where doubling the number of bacterial genera would decrease the precision at the genus level by ∼6%. Given this conservative assumption, the precision of WIsH with 1978 bacterial genera would approach 70%. Thus, with equal numbers of bacterial genera, we estimate that WIsH and our CRISPR spacers-based host prediction method would achieve similar levels of accuracy. In our opinion, a host predicted from spacers matching a viral contig arguably provides a more convincing case because of previously recorded phage–bacteria interactions in the CRISPR array. On the other hand, WIsH shows an association in terms of genomic composition between a phage and its putative host.

We also believe that the spacer database and pipeline developed here stand out for their user-friendly design. It requires a one-time download (database and script) and the users do not need to pre-select bacterial genomes for more accurate predictions. Furthermore, the predicted hosts are accompanied by the information on the filter that was used to predict the phage host. Therefore, since precision varies by filter, the user knows how reliable each prediction is and can make informed decisions about how the results are used. The use of a set of well-defined criteria offers a transparent and easy way to critically assess and interpret the host prediction of uncharacterized phages. Other spacer databases exist, such as the Integrated Microbial Genome/Virus (IMG/VR) system (38) and the CRISPRCasFinder website (24). The IMG/VR website provides a tool to blast single phage sequences against their Viral Spacer Database, using the *E*-value as a cut-off. However, none of these databases provide an easy system for linking spacer sequences to organisms and then automatically performing host predictions with optimized parameters. Previous studies that characterize the viromes of various environments have made phage host predictions with CRISPR spacers by building custom spacer databases (28,39–43). They used different sources (metagenome-derived spacers or spacers from deposited genomes), CRISPR identification programs and filters to assign hosts. To our knowledge, our spacer database, web platform and command-line program represent the first set of tools that automates, simplifies and standardizes phage host predictions based on CRISPR spacers from spacers identification to database construction and alignment filters.

## DISCUSSION

We optimized bacterial host predictions of unknown phages based on CRISPR spacers. By allowing at most two mismatches between a given spacer and a queried phage as well as following a set of criteria inspired by the biology of CRISPR–Cas systems, we obtained a recall of 49% and a precision of 69% at the genus level for our benchmark dataset. A set of tools was developed to streamline host prediction, including a spacer database that contains >11 million spacers, a web platform to explore the database using taxonomic filters and to perform predictions for single phage genomes, and a command-line tool that automates alignment and host predictions for larger virome datasets. We used criteria that were inspired by the way CRISPR arrays chronologically accumulate spacers and how Cas proteins recognize invading nucleic acids. This CRISPR spacer-based approach was shown to be particularly well suited for unknown phages from the gut virome. This host prediction method also distinguishes itself by its customizability and user-friendly interface to facilitate the interpretation of host prediction results. Despite showing high precision, our method is limited in its recall. One way to improve the recall may be to use our proposed method in conjunction with other host prediction tools, particularly those that rely on other prediction strategies. Mining spacers from bacterial metagenomes of the same sample used for viral metagenomics might increase the number of phages for which a prediction is obtained since such spacers likely reflect specific and recent interactions with unknown phages (39,41). However, using spacers from metagenomes involves identifying spacers from potentially misannotated bacterial contigs, which could lead to inaccurate host predictions. Additional work is likely needed to optimize host predictions with metagenome-derived spacers, possibly by incorporating other metrics so that precision is not compromised. The spacer database is currently restricted to bacterial spacers, but CRISPR–Cas systems are present in both archaea and bacteria. However, given the limited number of archaeal genomes, and more importantly their viruses (only 250 archaeal viruses that infect 23 hosts genera are publicly available), we could not provide accurate measurements of the host prediction for archaeal viruses. As more archaea and viruses get sequenced, a spacer-based host prediction method may be developed, tailored to this Domain.

The CRISPR spacer database expands the diversity of sequences available to find homology with unknown phages. Our comprehensive analysis led to the identification of the most performant and biologically relevant criteria, verified on thousands of phages, to predict the bacterial hosts of unknown phages. The first set of tools to automate bacterial host predictions based on CRISPR spacers was also developed, providing the scientific community with a website and command-line tool to perform high-quality, consistent and easy-to-interpret predictions. Altogether, this approach will contribute to a better characterization of unknown phages from viral metagenomics and a better understanding of their ecological role by revealing the hosts that they infect.

## DATA AVAILABILITY

Data analysis and visualization were conducted using scripts that were written in Python 3 in the Jupyter interactive environment. The command-line tool described in this study and the Jupyter notebook are available at https://github.com/edzuf/CrisprOpenDB. The CRISPR Spacer database is freely available at http://crispr.genome.ulaval.ca. The list of software and packages used in this study are available in Supplementary Table S3. Additional relevant data are available from the corresponding author on request.

The list of phages and their accession numbers used as a benchmark dataset is available in Supplementary dataset 1.

## Supplementary Material

gkab133_Supplemental_Files
