# Supplementary material for: Streamlining CRISPR spacer-based bacterial host predictions to decipher the viral dark matter
Source: Nucleic Acids Res. 2021 Mar 2;49(6):3127–38. doi: 10.1093/nar/gkab133 (PMC8034630; doi:10.1093/nar/gkab133)
Supplement: gkab133_Supplemental_Files [file gkab133_supplemental_files.zip › Supplementary Tables.docx]

Table S1: Performance of all possible combinations of filters

| Order of filters | Recall (%) | Precision (genus level, %) |
| --- | --- | --- |
| 1-2-3 | 49 | 69 |
| 1 | 46 | 62 |
| 2 | 81 | 41 |
| 3 | 61 | 22 |
| 1-2 | 48 | 68 |
| 1-3 | 49 | 68 |
| 2-1 | 82 | 42 |
| 2-3 | 99 | 35 |
| 3-1 | 70 | 35 |
| 3-2 | 73 | 32 |
| 1-3-2 | 49 | 69 |
| 2-1-3 | 82 | 42 |
| 2-3-1 | 99 | 35 |
| 3-1-2 | 70 | 35 |
| 3-2-1 | 75 | 35 |

1 = Number of mismatches (max = 2)

2 = Number of regions targeted

3 = Spacers positions within the CRISPR locus

Table S2: List of customizable parameters for the command-line tool

| Option | Description |
| --- | --- |
| -h, --help | Show help message. |
| -i INPUT, --input INPUT | Input file in FASTA or multi-FASTA format (required). |
| -m MISMATCH, --mismatch MISMATCH | Number of mismatches tolerated for the alignment. Must be between 0 and 5, default is 2. |
| -a ALIGNER, --aligner ALIGNER | Alignment tool to use. Options are blast or fasta36, default is blast. |
| -b BLASTDB, --blastdb BLASTDB | BLAST database to use for the alignment |
| -f FASTADB, --fastadb FASTADB | FASTA database to use for the alignment |
| -n NUM_THREADS, --num_threads NUM_THREADS | Maximal number of threads (CPUs) to use. Default is 1. |
| -r, --report | Show full report of host identification. Disabled if not specified. |
| -t, --table | Export result table with alignment results and spacers information in separate CSV file. Disabled if not specified. |
| -u, --unknown | Keep spacers with unknown genus for prediction. Disabled if not specified. |

Table S3: List of software and packages used in this study

| Step | Software/package | Link/Comment |
| --- | --- | --- |
| Download bacterial genomes |  | [ftp.ncbi.nlm.nih.gov/genomes/genbank/bacteria/assembly_summary.txt](ftp://ftp.ncbi.nlm.nih.gov/genomes/genbank/bacteria/assembly_summary.txt) |
| CRISPR Identification | CRISPRDetect v2.2 | <https://github.com/ambarishbiswas/CRISPRDetect_2.2>  -array_quality_score_cutoff 3 |
| SQL Database | Python packages:  -biopython  -sqlite3 v2.6.0  -ete3 v3.1.1 |  |
| Command line tool | blastn  Python packages:  -sqlite3 v2.6.0  -biopython v1.77  -pandas v1.0.1  -numpy v1.18.1 | -task ‘blastn’ |
| Download phage genomes |  | <https://www.ncbi.nlm.nih.gov/labs/virus/vssi/#/virus?SeqType_s=Nucleotide&VirusLineage_ss=Bacteriophage,%20all%20taxids> |
| Proof of concept | VIBRANT v1.2.1 | <https://github.com/AnantharamanLab/VIBRANT> |
|  | Shkoporov *et al.* 2019*,* human gut virome | <https://figshare.com/articles/The_human_gut_virome_is_highly_diverse_stable_and_individual-specific_/9248864> |
| Data analysis and visualization | Jupyter notebook  Python packages:  -sqlite3 v2.6.0  -collections  -matplotlib v3.1.3  -numpy v1.18.1  -plotly v4.1.1  -ete3 v3.1.1  -pandas v1.0.1  -scipy v1.4.1 |  |
